# Acute myeloid leukemia: from NGS, through scRNA-seq, to CAR-T. dissect cancer heterogeneity and tailor the treatment

**DOI:** 10.1186/s13046-023-02841-8

**Published:** 2023-10-06

**Authors:** Edoardo Peroni, Maria Luigia Randi, Antonio Rosato, Stefano Cagnin

**Affiliations:** 1 Immunology and Molecular Oncology Unit, Veneto Institute of Oncology, IOV-IRCCS, Padova, 35128 Italy; 2 First Medical Clinic, Department of Medicine-DIMED, University of Padua, Padua, Italy; 3 Department of Surgery, Oncology and Gastroenterology, University of Padua, Padua, Italy; 4 Department of Biology, University of Padova, Padova, 35131 Italy; 5 CIR-Myo Myology Center, University of Padova, Padova, 35131 Italy

**Keywords:** Acute myeloid leukaemia (AML), Next generation sequencing (NGS), Single cell sequencing (scRNA-seq), Intratumoral heterogeneity, Therapeutic targets, Personalized medicine

## Abstract

Acute myeloid leukemia (AML) is a malignant blood cancer with marked cellular heterogeneity due to altered maturation and differentiation of myeloid blasts, the possible causes of which are transcriptional or epigenetic alterations, impaired apoptosis, and excessive cell proliferation. This neoplasm has a high rate of resistance to anticancer therapies and thus a high risk of relapse and mortality because of both the biological diversity of the patient and intratumoral heterogeneity due to the acquisition of new somatic changes. For more than 40 years, the old gold standard “one size fits all” treatment approach included intensive chemotherapy treatment with anthracyclines and cytarabine.

The manuscript first traces the evolution of the understanding of the pathology from the 1970s to the present. The enormous strides made in its categorization prove to be crucial for risk stratification, enabling an increasingly personalized diagnosis and treatment approach.

Subsequently, we highlight how, over the past 15 years, technological advances enabling single cell RNA sequencing and T-cell modification based on the genomic tools are affecting the classification and treatment of AML. At the dawn of the new millennium, the advent of high-throughput next-generation sequencing technologies has enabled the profiling of patients evidencing different facets of the same disease, stratifying risk, and identifying new possible therapeutic targets that have subsequently been validated. Currently, the possibility of investigating tumor heterogeneity at the single cell level, profiling the tumor at the time of diagnosis or after treatments exist. This would allow the identification of underrepresented cellular subclones or clones resistant to therapeutic approaches and thus responsible for post-treatment relapse that would otherwise be difficult to detect with bulk investigations on the tumor biopsy. Single-cell investigation will then allow even greater personalization of therapy to the genetic and transcriptional profile of the tumor, saving valuable time and dangerous side effects. The era of personalized medicine will take a huge step forward through the disclosure of each individual piece of the complex puzzle that is cancer pathology, to implement a “tailored” therapeutic approach based also on engineered CAR-T cells.

## Introduction

Acute myeloid leukemia (AML) is an aggressive blood cancer characterized by an accumulation of immature cells of the myeloid lineage. Although most patients initially respond to chemotherapy, about 75% relapse and succumb to the disease within 5 years after diagnosis [1]. The latter aspect of the disease highlights how important it is to better understand the underlying mechanisms that prevent the elimination of all cancer cells to develop more targeted and efficient therapies.

In the 19th century Lewis Carroll wrote “Who in the world am I? Ah, that is the great puzzle.” This sentence referring to the famous novel “Alice’s adventure in wonderland” seems to fit perfectly in our scientific context about leukemia and, more generally, the development and evolution of cancer.

We could paraphrase the question written by Carrol in “What disease am I?”. Already in the end of the 1800s, the German pathologist David Paul Von Hansemann published the first observations on the state of cells that, losing their specific characteristics, assume an undifferentiated and atypical appearance. He was in fact the first to coin the terms anaplasia and dedifferentiation referring to the nuclear and mitotic differences that characterize morphological heterogeneity within the tumor tissue [2]. In the 1970s, a major work by Peter Nowell provided to the scientific community a milestone in reference to the clonal evolutionary model on tumor growth [3]. Neoplastic development is defined by a series of mutational events. These genetic lesions occur in somatic cells and first and foremost characterize the loss of proliferation control and programmed death regulation. These genetic alterations, as in Darwinian evolution, may grant selective advantages to these clonal subpopulations, allowing survival to chemo- or radio-therapy treatments, as well as the ability to evade the immune system and acquire metastatic features [4, 5].

The complex adaptive process of cancer evolution involves genetic assortment combined with clonal selection and subsequently subclonal expansion.

Certainly cancer is a great puzzle, and although the cytogenetic heterogeneity of leukemia cells has been recognized for more than 40 years [6], the enormous molecular heterogeneity of the disease was evidenced only with the advent of new massive sequencing techniques such as Next Generation Sequencing (NGS) [7]. It has also been clarified that some genomic alterations are shared by the entire tumor, but not all cancer cells show identical genomic and cytogenetic profiles. Interestingly, the single-cell analysis, now possible thanks to advanced miniaturization techniques, has shown that cancers are characterized by enormous cellular heterogeneity presenting different markers can be used for the diagnosis and treatment.

This review will discuss risk factors associated with AML, therapies developed to contrast the progression of AML, and recent discoveries based on genomic methods and single-cell analyses that could be the basis for new therapeutic approaches.

## AML: description of the pathology

AML is a biologically heterogeneous disease characterized by a broad category of overlapping hematologic aggressive neoplasms associated with rapid onset with a progressive course and often chemoresistant to cytotoxic therapies [8, 9]. Normally, the course of the disease begins in the bone marrow (BM) and then rapidly progresses to the bloodstream. It is not uncommon for leukemic cancer cells to invade other tissues and organs such as lymph nodes, liver, testes, spleen, and central nervous system (brain and spinal cord).

This blood cancer is characterized by malignant transformation of progenitor/precursor cells committed to the myeloid lineage. The blasts fate is then compromised, impairing maturation and differentiation into granulocytic, monocytic, erythroid and/or megakaryocytic elements. The result of abnormal and poor differentiation of myeloid cells is also the dangerous accumulation of high levels of immature malignant cells and the decrease of normally differentiated blood components in blood and non-blood tissues [10, 11].

Clinical manifestations of AML include symptoms and signs associated with cytopenia, for example, anemia, infections related to low white blood cell counts, as well as bleeding or ecchymosis due to thrombocytopenia. In parallel, they may be accompanied by constitutional symptoms, such as abnormalities of metabolic pathways and various complications such as fever, bone pain, shortness of breath, pale skin, lethargy, and fatigue. AML has also been called acute myeloid leukemia and acute non-lymphocytic leukemia [12, 13].

### Leukemogenesis: risk factors

AML is the result of a series of mutational events that occur in hematopoietic stem cells (HSCs) that include genetic and epigenetic alterations effectively altering normal hematopoiesis. Indeed, the leukemic condition appears to be maintained by an uncommon subpopulation of leukemic cells called leukemic stem cells (LSC), also referred as leukemia-initiating cells (LIC). These cells, identified at the end of the last century, are fully transformed by driver mutations that confer typical characteristics of stem cells and therefore the ability to reconstitute the heterogeneous condition of leukemia, self-renewal ability and drug resistance [14, 15]. In most cases it is still not possible to deduce the exact event that causes AML. In any case, it is now established that the disease can develop thanks to the contribution of various aspects that identify various acquired risk factors: (a) the age and therefore the senescence of HSCs; (b) exposure to anticancer therapy with chemotherapy or ionizing radiation (the total or part loss of chromosome 5 or 7 following treatments with alkylating agents and/or radiotherapy as well as the mutagenic action of topoisomerase 2 inhibitors is well documented [16]); (c) obesity; (d) smoking [17, 18]; (e) family predisposition (Table 1). Family predisposition can be due to aneuploidy as trisomy 21, Fanconi’s anemia and some germline mutations in Tumor Protein P53 (*TP53*), CCAAT Enhancer Binding Protein Alpha (*CEBPA*), ETS Variant Transcription Factor 6 (*ETV6*), Ankyrin Repeat Domain Containing 26 (*ANKRD26*), DEAD-Box Helicase 41 (*DDX41*), RUNX Family Transcription Factor 1 (*RUNX1*), Telomerase RNA Component (*TERC*), GATA Binding Protein 2 (*GATA2*), Signal Recognition Particle 72 (*SRP72*), and Telomerase Reverse Transcriptase (*TERT*) genes [19, 20]; (f) acquired somatic genetic lesions that will examined through the process of leukemogenesis.

**Table 1:** Risk factors concerning AML development

Predisposing factors of AML	
Antecedent blood disorder	- Myelodysplastic syndromes - Chronic Myeloid Leukemia - Polycytemia Vera - Essential Thrombocytemia - Paroxismal Nocturnal - Hemoglobinuria - Aplastic Anemia - Myelofibrosis
Genetic syndromes, family predisposition and congenital genetic lesions	- Bloom Syndrome - Fanconi Anemia - Kostmann Syndrome - Wiskott-Aldrich Syndrome - Ataxia-teleangiectasia - Down Syndrome - Klinefelter Syndrome - Patau Syndrome - Germline mutations
Chemotherapy drugs	- Alkylating agents: cyclophosphamide, melphalan, nitrogen mustard - Topoisomerase II inhibitors: etoposide, teniposide - Chloramphenicol - Phenylbutazone - Chloroquine - Methoxypsoralen
Environmental factors and lifestyle	- Radiation exposure - Benzene - Smoking - Alcohol use - Dyes - Herbicides - Pesticides - Obesity
Acquired genetic mutations	- Founder mutations - Driver mutations

**Fig. 1:**
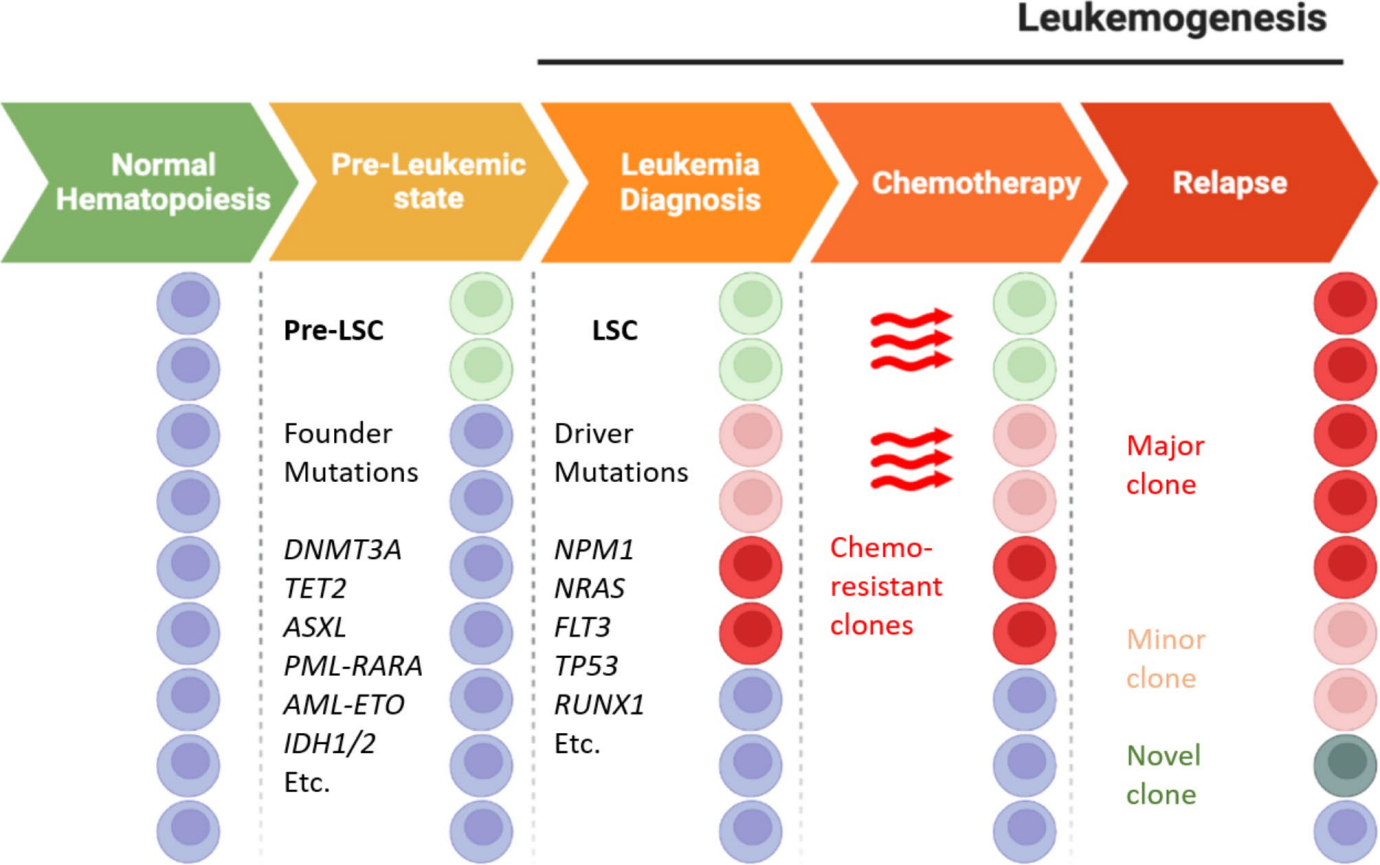
Leukemogenesis is a multistep process in which normal hematopoiesis is impaired by the accumulation of preliminary mutational events, founder mutations, which characterize the Pre-SLC state. The SLC condition develops following mutational events defined as driver mutations which can give rise to clones resistant to anticancer treatments and therefore responsible for the relapse of the disease (figure edited with biorender.com)

Leukemogenesis can certainly be defined as a multistep process (Fig. 1) characterized by the succession of the acquisition or loss of genetic alterations which will then define the heterogeneity that characterizes the disease. In fact, during life HSCs undergo some genetic insults that lead to the acquisition of mutations, called founder mutations, which however are not a sufficient condition for the development of AML but rather to a pre-leukemic condition of cells defined as preleukemic stem cell (pre-LSCs) [21, 22].

Founder mutations mostly affect genes involved in epigenetic regulation. It has in fact been documented that the first mutational events compromise the correct functionality of DNA methyltransferase 3a (DNMT3A), isocitrate dehydrogenases 1 and 2 (IDH1/2), ten-eleven translocation 2 (TET2) and ASXL transcriptional regulator 1 (ASXL1) compromising therefore the maturation of blasts and promoting the ability of self-renewal and clonal expansion [23, 24]. When a pre-LSCs unfortunately undergoes a mutational event responsible for the genesis of AML, this is defined driver mutation. *De facto*, these genetic lesions occur temporally following the founder mutations [25], and confer proliferative advantages to tumor cells by impairing normal apoptotic activity compromising the function of FMS-like tyrosine kinase 3 (FLT3) [10], neuroblastoma RAS viral oncogene homolog (NRAS) [26], nucleophosmin (NPM1), and TP53 [27, 28]. Leukemogenesis is hence a process that involves the deregulation of several pathways. Genes commonly mutated in AML can be grouped into 8 different categories which include 1) genes involved in signal transduction 2), tumor suppressors, 3) genes responsible for DNA methylation and 4) chromatin modifications, 5) myeloid transcription factors, 6) nucleophosmin, 7) genes related to the spliceosome complex, and 8) to the cohesine complex (Table 2).

## How to classify AML: AML subtypes

Over the years, different classification schemes have been developed for all the various forms of acute myeloid leukemia. This because the development of new molecular biology based techniques for the leukemic cells classification: microarray gene expression, DNA and RNA sequencing. There are 3 major systems that are used to classify AML into subtypes. The French-American British classification was in use earlier and has been replaced by the newer World Health Organization classification and in parallel with the guidelines of the European Leukemia Network.

The **French-American-British** (FAB) classification was developed by a group of French, American, and British leukemia experts in 1976s and it has been used up to 2001 [29]. This classification included the division of AML into eight subtypes (from M0 to M7) in relation to the origin and the level of maturity of cell from which the leukemia developed. In detail, M0 to M5 subtypes comprise hematological disorders that originate from immature white blood cells. AML M6 subtype has its beginning from very immature red blood cells, while the subtype M7 originates from immature megakaryocytes [30]. Subsequently, over the years, changes have been made to the FAB classification thanks to the discovery of various genetic factors involved in the pathology (Table 3).

However, more recent classification schemes, based on molecular and prognostic scoring systems, have replaced the FAB classification.

After the advent of the **World Health Organization** (WHO) classification in 2001, AML were classified according to this more modern system, revised in 2008 with the 4th edition [31] and then in 2016 with the 5th edition [32].

If FAB classification has some problems because it does not take into consideration various factors that may affect the prognosis, WHO classification integrates different categorizing aspects such as clinical, morphologic, immunophenotypic, and genetic features. This approach was taken in an attempt to define clinically relevant biological entities in order to incorporate new knowledge about these disorders to gain a better patient perspective [8]. WHO then proposed a newer system to classify AML with minimum of 20% of blasts in BM or blood to diagnose AML (FAB classification requires 30% of blasts). An important distinction was to detach de novo AML, from secondary AML developing from myelodysplastic syndrome, as well as subtypes of therapy related disease.

Many of these revisions involved prognostication derived from gene expression analyses and next generation sequencing studies according whose six categories of AML were identified: (1) categories encompass AML with recurrent genetic aberrations, (2) AML with myelodysplasia related changes, (3) therapy related myeloid neoplasms, (4) AML not otherwise specified (NOS), (5) myeloid sarcoma, (6) myeloid proliferations related to Down Syndrome. Moreover, a new category, the myeloid neoplasms with germline predisposition, was added to incorporate the subgroup of familial AML associated with germline mutations (e.g., germline *RUNX1, GATA2, CEBPA*) (Table 3).

In 2022, the **European Leukemia Network** (ELN-Net) updated the 2017 recommendations for the diagnosis and management of AML in adults [9]. This revision aimed to correlate genetic abnormalities with clinical variables and prognostic impact, leading to the reclassification of risk stratification. The ELN-Net continued to group patients into three risk categories: favorable, intermediate, and adverse risk. However, certain changes were introduced to reflect new insights into AML disease biology and risk assessment [33].

One significant change in the ELN2022 classification is the categorization of *CEBPA* mutations as favorable risk. Now, *CEBPA* mutations are restricted to in-frame mutations in the basic leucine zipper (bZIP) region, whether they occur as mono- or biallelic mutations (Table 3). Additionally, the previous division based on high or low *FLT3-ITD* allelic ratio (AR) has been abandoned. All patients with *FLT3-ITD* are now placed in the ELN2022 intermediate risk group, irrespective of the presence or absence of an *NPM1* mutation. *NPM1* mutations continue to indicate favorable outcomes, except when co-occurring with adverse risk cytogenetics, which now places them in the ELN2022 adverse risk group. Furthermore, the definition of a complex karyotype has been updated to exclude hyperdiploid karyotypes with multiple trisomies from this group.

In the ELN2017 classification, mutations in *ASXL1*, *RUNX1*, and *TP53* were introduced as new adverse risk prognostic factors. Now, in ELN2022, so-called myelodysplasia-related gene mutations in *BCOR, EZH2, SF3B1, SRSF2, STAG2, U2AF1*, or *ZRSR2* are additional markers defining ELN2022 adverse risk, when not accompanied by favorable risk genetics. Moreover, a 10% variant allele frequency (VAF) threshold for *TP53* mutations has been established to assign individuals to ELN2022 adverse risk [9].

**Table 2:** Genes involved in the development of AML and their functional categories

Functional categories	Consequences of genetic alterations
Signal transduction genes	Mutations in *FLT3*, a class III tyrosine kinase receptor, or *NRAS, KRAS, c-KIT, PTPN11* confer proliferative advantages through hyperactivation of related signal cascades such as JAK/STAT, PI3K/AKT/NFKB and RAF/MEK/ERK.
Myeloid transcription factors (TF)	Mutations in myeloid TF (*AML1, CEBPA, RUNX1*) or genetic fusions following chromosomal rearrangements t(8;21)(q22;q22) *RUNX1-RUNX1T1, AML1-ETO*, t(15;17)(q24;q21) *PML-RARA*, or inv(16) (p13.1q22) and t(16; 16)(p13.1;q22) *CBFB-MYH11*, are events responsible for transcriptional deregulation and affect correct hematopoiesis.
Tumor Suppressor Genes	Mutations in *P53*, *WT1* or *PHF6* can deregulate normal transcription activity and alter checkpoints responses of the cell (for instance mutated P53 blocks the degradation of PTEN increasing proliferative action)
Spliceosome Complex	Mutations in components of the spliceosome complex such as *SRSF2*, *SF3B1, U2AF1* and *ZRSR2* can alter proper mRNA maturation with intron retention or exon skipping events.
Multifunctional shuttling protein	Mutations in nucleophosmin (*NPM1*) are responsible for its anomalous cytoplasmic localization and consequently of the proteins that interact with it, for instance ARF, influencing the ribosome biogenesis and P53 stability.
Cohesin Complex	Mutations in *SMC1A, SMC3, STAG2, RAD21* affect normal chromosomal segregation and transcriptional regulation by increasing chromatin accessibility and increasing the binding of TF AML1 and GATA2 with consequent proliferative increase and decrease in cell differentiation.
DNA methylation genes	Mutations in genes involved in DNA methylation such as *DMNT3A, TET2, IDH1/2* can alter epigenetic regulation
Chromatin modifiers	Epigenetic homeostasis can be perturbed in relation to mutations in genes involved in chromatin modifications; for instance *ASXL1, EZH2, MLL* partial duplication or t(9;11)(p22;q23) *KMT2A-MLLT3* alias *MLL-AF9* gene fusions.

**Table 3:** Evolution of AML tabulation from the 1970s with the advent of FAB classification method and the following development of WHO and ELN classification. Table improved and revised from [20]

Classification schemes for acute myeloid leukemia (AML)
**French-American-British (FAB) classification**
1. AML subtypes were defined on the basis of morphologic features and cytochemical methods, categorized as M0 through M7 • **M0-M5**: include leukemia involving myeloid blasts with varying degree of maturation • **M6**: acute erythroid leukemia • **M7**: acute megakaryocytic leukemia • **M3**: represents the distinct subtype of acute promyelocytic leukemia (APL) 2. FAB criteria evidenced M2 (25%) and M4 (20%) as the most common subtypes disease
**World Health Organization (WHO) classification**
1. **AML with recurrent genetic abnormalities** • AML with t(8;21)(q22q22.1); *RUNX1-RUNX1T1* • AML with inv(16)(p13.1q22) or t(16;16)(p13.1;q22); *CBFB-MYH11* • APL with *PML-RARA* • AML with t(9;11)(p21.3;q23.3); *KMT2A-MLLT3* • AML with t(6;9)(p23;q34.1); *DEK-NUP214* • AML with inv(3)(q21.3q26.2) or t(3;3)(q21.3;q26.2); *GATA2, MECOM* • AML (megakaryoblastic) with t(1;22)(p13.3;q13.1); *RBM15-MKL1* • Provisional entity: AML with *BCR-ABL1* • AML with mutated *NPM1* • AML with biallelic mutation of *CEBPA* • Provisional entity: AML with mutated *RUNX1* 2. **AML with myelodysplasia-related changes** 3. **Therapy-related myeloid neoplasms** 4. **AML, not otherwise specified (NOS)** • AML with minimal differentiation • AML without maturation • AML with maturation • Acute myelomonocytic leukemia • Acute monoblastic and monocytic leukemia • Pure erythroid leukemia • Acute megakaryoblastic leukemia • Acute basophilic leukemia • Acute panmyelosis with myelofibrosis 5. **Myeloid sarcoma** 6. **Myeloid proliferations associated with Down syndrome** • Transient abnormal myelopoiesis (TAM) associated with Down Syndrome • Myeloid leukemia associated with Down Syndrome
**European Leukemia-Net (ELN) classification**
1. The **favorable** category includes: • Core binding factor AML, defined by the cytogenetic abnormalities t(8;21)(q22;q22.1) *RUNX1-RUNX1T1*; inv(16)(p13.1q22) or t(16;16)(p13.1;q22) *CBFB-MYH11*; • It is implicated also the favorable prognosis of AML with *NPM1* or biallelic mutated *CEBPA*, disregarding the concomitant presence of gene mutations. • Similar favorable impact noticed of *NPM1*; in frame mutations of basic basic leucine Zipper (bZIP) region of CEBPA 2. The **intermediate** classification includes: • Those cytogenetic and/or abnormalities not classified as favorable or adverse • Mutated *NPM1* in presence of *FLT3-ITD* • Wild type *NPM1* with *FLT3-ITD* (without adverse-risk genetic lesions • t(9;11) (p21.3;q23.3), *MLLT3-KMT2A* aberrations 3. The **adverse** risk subtype includes: • The high risk mutation *TP53* (with VAF ≥ 10%) and mutations in *RUNX1, ASXL1, EZH2, SF3B1, SRSF2, STAG2, U2AF1* and *ZRSR2* (not coexisting with favorable risk subtypes) • Wild type *NPM1* with *FLT3-ITD* high allelic ratio (> 0.5) carries a poor prognosis and it is also comprised in the adverse risk group • Monosomal or complex karyotypes (≥ 3unrelated chromome bnormalities) • Specific cytogenetic markers of high risk disease including t(6;9)(p23;q34.1) / *DEK-NUP214*, t(v;11q23.3) / *KMT2A* rearranged, t(9;22)(q34.1;q11.2) / *BCR-ABL1*, inv(3)(q21.3q26.2) or t(3;3)(q21.3;q26.2), *GATA2*, *MECOM*(*EVI1*), monosomy 5 or del(5q), monosomy 7, − 17 / abn (17q)

### Therapies used to contrast AML evolution

*Standard therapies.* In the United States, the incidence of AML is 4.3 per 100,000 people with an average age at diagnosis of 68 years and similar statistics were also found in Europe with 3.7 per 100,000 people [34]. Among all subtypes of leukemia, AML is the one with the highest percentage of poor prognosis, accounting for about 62% of cases according to SEER database [35]. Although about 50 years ago AML was considered an incurable disease, today about 35–40% of cases under the age of 60 have a favorable prognosis while unfortunately remains dismal with a cure rate of around 5–15% for geriatric patients [36]. In general, little has changed regarding the therapeutic approach to AML for decades. In fact, the treatment involves a first chemotherapy approach called **induction therapy** while the post-remission strategy involves consolidation therapy with intensive chemotherapy treatment as well as hematopoietic stem cell transplantation (HSCT).

Induction therapy provides the typical “7 + 3” treatment defined in this way as it involves the continuous infusion of Cytarabine (Ara-C) for 7 days combined with 3 days of Anthracycline (Idarubicin, Daunorubucin, Mitoxantrone) [37, 38]. This therapeutic approach has a fairly good response in patients younger than 60 years, reaching a complete remission between 60% and 85% of cases, however capitulating considerably in patients over 65 years where the response rate to therapy is about 50% and complete remission (CR) of approximately 10% of cases [36]. This is likely due to the fact that older patients may have collected a greater number of genetic and cytogenetic abnormalities, as well as the comorbidity of clinically significant conditions. An example is given by genetic lesions in TP53, the most important tumor suppressor protein that regulates cellular response to various stresses, which lead to greater resistance to chemotherapeutic agents with consequent tumor progression and poor prognosis [39]. It was also attempted to assist the chemotherapy action with humanized CD33 antibodies such as Gentuzumab Ozogamicin (GO), obtaining significantly improved event-free survival of newly diagnosed AML pediatric patients or whose with favorable and intermediate cytogenetic-risk disease but poor results with those with adverse-risk [40, 41].

As mentioned above, post-remission strategies involve **consolidation treatment** with intensive chemotherapy and allogenic HSCT.

The first gives good results in patients under the age of 60 and with more favorable ELN genetic profile with a rate cure that is between 60 and 70%. This treatment involves a variable regimen between 2 and 4 cycles of Ara-C with an intermediate dose (IDAC) between 1000 and 1500 mg/m^2^. Clinical studies have shown that increasing the dose of Ara-C for the treatment of AML has no clinical findings [42], although the balance with better results between the number of cycles such as the optimal dose of chemotherapy remains an open discussion. Unfortunately, as extensively treated, patients with unfavorable genetic and cytogenetic characteristics, as well as the presence of other risk factors such as age and the presence of other pathologies, do not show benefits with this type of therapy, indeed the risks tend to be greater than the benefits.

Allogenic HSCT provides the most incisive effects against cancer thanks to the immunologic anti-leukemic graft versus leukemia effect [43]; AML is in fact the most frequent indication for transplantation [44, 45]. It benefits those patients in whom, regrettably, minimal residual disease (MRD) monitoring shows incomplete remission with conventional chemotherapy approaches [46, 47]. Likewise, in this case, geriatric patients present problems in the implementation of the transplant, and in fact only a small fraction of them are eligible by carefully balancing the relationship between risks and benefits. Normally allogeneic HSCT is indicated in those patients whose risk of relapse is between 35 and 40% and is the only treatment option for patients with primary refractory disease [48] (Table 4).

**Maintenance therapy** is the third phae of treatments, and it is intended to reduce the risk of recurrence in patients who have achieved CR following remission with intensive chemotherapy treatment. While this treatment approach lacks a universal definition, the FDA characterizes it as an extensive but limited and less toxic treatment regimen [9]. In the past 30 years, the approach to maintenance therapy in AML has shifted from using chemotherapeutic agents to employing targeted therapies and enhancing immune system modulation [49]. When devising maintenance therapy for AML, it is crucial to consider potential additional toxicities and the patient’s quality of life. An ongoing trial at Md Anderson Cancer Center is exploring these considerations, employing a genomics-driven approach to study different combinations of oral maintenance therapy in AML (NCT05010772) [50]. This trial involves adult patients in first remission, who will receive various oral decitabine-based regimens for up to 24 cycles, based on their specific induction therapy and transplant eligibility. It is now recommended maintenance therapy for all AML patients through ongoing clinical trials. In cases of CBF-AML, parenteral decitabine is advised when designated cycles of certain regimens cannot be completed or when persistent molecular MRD is observed. For intermediate and adverse-risk AML, HSCT is preferred, followed by appropriate maintenance. For patients unable to proceed to HSCT, a combination of HMA +/- venetoclax is suggested. In patients with targetable mutations, corresponding inhibitors are continued as maintenance therapy after remission induction.

Over the past 25 years, the approach to AML maintenance has evolved from low-intensity chemotherapy to targeted therapies and immunotherapy. While high-risk AML cases generally benefit from maintenance therapy, ongoing trials will determine its potential benefits for less adverse-risk AML. It is important to consider the possibility of exacerbating genomic instability and clonal escape when designing such regimens.

**Table 4:** Summary scheme of anticancer therapy in relation to the type of patient whether or not eligible for intensive chemotherapy treatment

Patients eligible for intensive chemotherapy	
**Induction Therapy**	
Induction therapy “7 + 3” treatment (no age limit)	3 days of Anthracycline (Daunorubicin 60 mg/m^2^ or Idarubicin 12 mg/m^2^ or mitoxantrone 12 mg/m^2^) and 7 days of continuous induction of Ara-C (100–200 mg/m^2^)
*FLT3* mutated patients	Addition to “7 + 3” regimen of FLT3 inhibitor Midostaurin and continued for at least 1 year after consolidation therapy
CD33-positive patients	Addition to “7 + 3” regimen of GO recommended for favourable and intermediated genetic risk
Patients with therapy related AML	CPX-351 gives better results compared to “7 + 3” regimen
Elderly and adverse risk patients	Clinical trials and investigation therapy are encouraged
**Consolidation Therapy**	
Favourable genetic risk	Intermediate or high dose of Ara-C (1000–3000 mg/m^2^) every 12 h for 3 days per 2 to 4 cycles.
Intermediate genetic risk	IDAC (1500 mg/m^2^) every 12 h for 3 days per 2 to 4 cycles.
	HDAC (3000 mg/m^2^) and autologous HSCT (in relation to individual risk of relapse, performance status, comorbidities, and patient preference)
Adverse genetic risk	Allogenic HSCT from matched-related or unrelated donor with same individual condition of autologous HSCT
**Patients NOT eligible for intensive chemotherapy**	
Ara-C	Low dose Ara-C (20 mg/m^2^) every 12 h for 1 to 10 days (not recommended for adverse genetic risk patients)
Hypomethylating agents	Azacitidine (75 mg/m^2^) for 1 to 7 days
	Decitabine (20 mg/m^2^) for 1 to 5 days
Antibody drug conjugate	GO if favourable or intermediate genetic risk patients CD33 positive
Best supportive care	Hydroxyurea for those patients with therapy related side effects
Investigation therapy	Clinical trials and investigation therapy are strongly encouraged

*New perspectives.* Thanks to the advent of new broad-spectrum molecular investigation technologies such as NGS, the evidence of the heterogeneity that characterizes almost all diseases, and in particular AML, has made it possible to overcome the “one-size-fits-all” concept typical of the old chemotherapeutic approach, which led to a low response rate invalidating the restoration of normal hematopoiesis being toxic to HSCT even in AML with good prognosis.

Now, by reason of the discovery of various genetic factors characterizing the disease, it is possible to use them as therapeutic targets both with inhibitory drugs and/or immunotherapeutic treatments already present and approved for AML or other diseases. The characterization of cellular heterogeneity therefore allows the development of further drugs and cell therapies that can treat the patient more appropriately, overcoming potential side-effects.

From some years, and increasingly in the future, the focus has been on a therapy tailored to the patient according to the molecular characteristics that differentiate the same disease in different patients.

These new therapeutic targets include (a) suppressor and oncogenic proteins target therapies, (b) protein kinase inhibitors, (c) epigenetic modulators, (d) chemotherapeutic agents, (e) mitochondrial inhibitors, (f) antibodies and immuno-therapies, (g) therapies that target the microenvironment that supports the maintenance and expansion of leukemic cells.

Genetic alterations of **FMS-Like Tyrosine Kinase 3** (*FLT3*) characterize about 25–30% of AML cases [51] as well as several solid tumors such as lung adenocarcinoma and gastrointestinal cancer [52]. 2017 and 2018 were 2 prolific years for FLT3 inhibitors since the first generation inhibitor Midostaurin, and then the next generation inhibitor Gilterinib, were approved by the US Food and Drug Administration (FDA).

Midostaurin was approved following the good results obtained from the CALGB10603 or RATIFY study published by Stone in 2017 [53–55] which showed a significant 7.2% increase in OS in relation to addition of Midostaurin to induction and consolidation therapy followed by a year of Midostaurin maintenance therapy. This small molecule with inhibitory activity has been shown to be effective with up-front AML targeting both AML with internal tandem duplication (*FLT3-IDT*) and those carrying mutations in the tyrosin kinase domain (*FLT3-TKD*).

Gilterinib received FDA approval at the end of 2018 when the ADMIRAL study published the results of the efficacy of this next generation inhibitor against r/r *FLT3-ITD* AML increasing OS by 9.3 months [56, 57]. It should also be mentioned how first generation inhibitors such as Sunitinib (SU11248), Midostaurin (PKC412), lestaurtinib (CEP-701), and Sorafenib (BAY43-9006) have initiated the development of new generation inhibitors such as Gilterinib, Crenolanib or Quizartinib[58, 59]. The inhibitory action of monoclonal antibodies such as LY3012218 [60], a novel bispecific antibody IgG-based FLT3xCD3 for the treatment of AML [61], is also studied.

Mutations in **Isocitrate dehydrogenase 1 and 2** (*IDH1* and *IDH2*) were found in approximately 7–14% and 8–19% of patients with AML respectively [62] as well as in solid tumors such as glioma, chondrosarcoma and cholangiocarcinoma [63]. Impairment of the correct function of IDH1/2 leads to the accumulation of the oncometabolite R-2-hydroxyglutarate with consequent epigenetic alterations that affect correct hematopoiesis. In 2017 and 2019 the US FDA approved Enasidenib AG-221 for IDH2 mutated AML in adult relapsed/refractory (r/r) patients, and Ivosidenib AG120 in patients with IDH1 mutations over 75 years of age or not eligible for standard chemotherapy treatment.

For both, data have been published that attest to an overall response rate (ORR) of 40% with an average increase in overall survival (OS) of 9 months [64, 65]. However, both show adverse effects associated with differentiation syndrome such as dyspnea, fever, pulmonary infiltrates and hypoxemia [64, 66].

Mutated *IDH* patients treated with a combinatorial regimen with Azacytidine together with Venetoclax [67] appear to have good prospects.

#### B-Cell Lymphoma 2

(BCL2) is a mithocondrial key regulator responsible of cell survival [68] which has proved to be a promising target in various neoplasms whose inhibition reactivates the apoptotic process [69, 70]. Venetoclax was highlighted as a promising drug for the treatment of r/r AML with an ORR of 19% [71, 72]. Further trials have then demonstrated the efficacy of the antitumor action of Venetoclax combined with other therapeutic agents. For example, in the NCT02203773 study a CR of 54% was found in AML patients treated with Venetoclax in combination with Decitabine or Azacytidine [73]. Encouraging results were also detected from the combination with low dose Ara-C (LDAC). In fact, the NCT02287233 study showed that 21% of patients over 60 years of age ineligible for classic chemotherapy [74] achieved excellent CR rates. Over the years, other combinatorial approaches have also been tried, assisting the synergistic action of different drugs. An example is a study with Trametinib, an inhibitor of the MEK tyrosine kinase, that stopped in phase 2 because data published in June of this year unfortunately did not show significant improvements in the pathological condition [75].

It is now evident how aberrant activation of **Hedgehog** (HH) signaling pathway is associated with a wide variety of neoplasms [76] promoting growth, migration and stemness. Furthermore, the expression of the transcription factor Glioma-associated oncogene homolog 1 (GLI) and Smoothened (SMO) are connected to the promotion of treatment resistance survival in AML [77, 78]. Current treatment strategies aim to inhibit GLI signaling by targeting SMO in cancer cells. Glasdegib is a SMO inhibitor approved by the FDA in 2018 for the treatment of AML in combination with LDAC. Data from the phase 3 study showed a significant improvement in patients over 75 years old ineligible for standard intensive chemotherapy who achieved a remission of 17% versus 2.3% and an OS of 8.3 versus 4.3 months when the treatment is done with LDAC only [79, 80].

Further in vitro research lines have revealed that synthetic Benzimidazole Mebendazole (MBZ) mediates its anti-leukemic effects via the inhibition of HSP70/90 chaperone activity and the promotion of the degradation of GLI transcription factors by the proteasome pathway [81].

#### Tumor Protein P53

(TP53) is a crucial tumor suppressor that maps to the short arm of chromosome 17 [82]. De novo *TP53* mutation or deletions are quite rare, about 10% in newly diagnosed AML, while they are more common in secondary therapy-related AML [10]. The range of mutations is very heterogeneous and often in conjunction with other genetic alterations involved in epigenetic regulation: *DNMT3A* and *TET2*, RAS / MAPK signaling (*NF1, KRAS / NRAS, PTPN11*), and RNA splicing (*SRSF2*) [83]. These genetic alterations are responsible for the resistance to DNA damaging chemotherapy agents [84].

Significant progress has been made by restoring the functional activity of TP53 with the agent Eprenetapopt (APR-246) in combination with Azacytidine, reaching phase 3 of the NCT03072043 clinical trial yielding high rates of clinical response and molecular remissions [85].

There are also encouraging data regarding clinical trial NCT04214860 that is in phase 1. Triplet regimen with Eprenetapopt, Venetoclax and Azacytidine demonstrated highly encouraging efficacy showing CR rate of 37% [86].

### Novel agents for the treatment of AML

#### Mouse Double Minute proteins

(MDMs) are E3 ligase that negatively regulate TP53 [87] via its proteasomal degradation. MDM2 is a potential therapeutic target investigated in the MIRROS clinical trial (NTC02545283) by administering the MDM2 antagonist molecule Idasanutlin to patients with r/r AML concomitantly with Ara-C treatment.

Published data show that the addition of Idasanutlin, despite significantly improving the ORR from 22.0 to 38.8%, proved to be ineffective in increasing the OS or CR rates in patients with r/r AML [88].

Another recently published study showed that *DNMT3A*-mutant samples had an overexpression of *MDM4*. ALRN-6924 a MDM2/4 inhibitor impairs the growth of *DNMT3A*^WT/R882X^ cells by inducing cell cycle arrest through the upregulation of TP53 target genes [89].

An additional potent MDM2 inhibitor is APG-115 that is being tested for patients with solid tumors, and has shown an optimal antileukemic activity in vitro and in xenograft models, both alone and synergically with standard-of-care hypomethylating agents Azacytidine and Decitabine, or the DNA-damaging agent Ara-C [90].

Another enzyme involved in the phosphorylation of TP53 and in the assembly of the mitotic spindle is the serine-threonine **Aurora Kinase** whose expression levels were found to be elevated in leukemic cells [91, 92].

In vitro studies have shown an increase in apoptosis and aneuploidy following anomalies in the formation of nuclei when Aurora kinase was inhibited using the small molecule antagonist MLN8054 [93].

This enzyme has thus emerged as a hypothetical therapeutic target against which several inhibitors such as Alisertib (MLN8237), Barasertib (AZD1152) have been developed. However, clinical studies both in single therapy and in combination with other therapeutic agents such as Ara-C and idarubicin have yielded discordant results [94, 95].

Promising in vitro results have been published about the cytotoxicity of these inhibitors in leukemic cells carrying the 8;21 translocation with *AML1-ETO* gene fusion [96].

#### Polo-Like Kinase

(PLKs) is a protein found to be overexpressed in AML whose inhibition in preclinical studies has shown to promote the arrest of the cell cycle and the reactivation of the apoptotic process [97, 98].

Among the various PLK inhibitors, volasertib has been studied most extensively in the clinic. Although phase 2 results of a clinical trial demonstrated improved OS in newly-diagnosed unfit AML patients through co-administration of volasertib to LDAC, it was not possible to confirm results in a larger subsequent phase 3 trial [99].

The Phase 1 dose escalation trial of Volasertib in combination with Decitabine has instead shown to have good results with an ORR of 23% and relative tolerated side effects from patients [100].

Onvansertib (NMS-P937) is another PLK1 inhibitor. It has a shorter half-life than Volasertib, which exhibited antitumor activity in both solid and hematologic cancer models including AML xenografts. For the moment, it has been shown to have synergistic activity with Ara-C in vitro [101].

Aberrant action of the methyl transferase **Disruptor of Telomeric Silencing 1-Like** (DOT1L) occurs following rearrangements in the long arm of chromosome 11 which occurs in about 2–11% of AML cases [102, 103] and promotes the overexpression of some genes involved in the leukemogenesis including *HOXA9* and *MEIS1* [104–106]. It is therefore a good target candidate. In fact, inhibitory molecules have been created such as Pinometostat (EPZ-5676) and multi-kinase inhibitor Sorafenib whose combined action has been shown to have significant cytotoxic levels in vitro both in cell lines and primary cell cultures from pediatric AML patients [107].

SNDX-5613 and KO-539 are small inhibitory molecules still in the primeval phase of study (NTC04067336, NTC04065399) which appear to have promising therapeutic effects and that have been granted orphan drug designation by the FDA for treatment of r/r AML. Published data show an ORR of 55% for SNDX-5613 [108–110].

Recently, Heikamp and colleagues demonstrated that mouse leukemia cell lines driven by *NUP98-HOXA9* and *NUP98-JARID1A* fusion oncoproteins are sensitive to the menin-MLL1 inhibitor VTP50469 by significantly increasing the OS of mice engrafted with cells from leukemia patients with genetic rearrangements above [111]. Table 5 summarizes possible therapeutic interventions against AML and perspectives and novel agents for the AML treatment.

**Table 5:** Summary table of the various anticancer treatments ranging from the suppression of oncogene expression to therapies to control the tumoral niche and micro-environment. Table revised and improved from [9]

AML therapeutic interventions, new perspectives and novel agents	
Suppressor or oncogenic proteins target therapies	• TP53 restoring agents Eprenetapopt APR-246 • MDMs inhibitors Idasanutlin, ALRN-6924 and APG-115 • Fusion transcripts targeting • EVI1 targeting • NPM1 targeting • Hedgehog inhibitors Glasdegib and Benzimidazole Mebendazole
Protein kinase inhibitors	• FLT3 I generation inhibitors (midostaurin, sunitinib, lestauritinib, safarenib) • FLT3 II generation inhibitors (quizartinib, gilteritinib, crenolanib, LY3012218) • MEK inhibitor Trametinib • KIT inhibitors • PI3K/AKT/mTOR inhibitors • Aurora inhibitors (MLN8054, Alisertib MLN8237, Barasertib AZD1152), CDK4/6 Inhibitors, CHK1, WEE1, and MPS1 inhibitors • Polo-like kinase (PLKs) inhibitors (Volasertib, Onvasertib NMS-P937) • SRC and HCK inhibitors
Epigenetic modulators	• New DNA methyltransferase inhibitors SGI-110, DOT1L inhibitors Pinometostat EPZ-5676, SNDX-5613, KO-539 • Menin-MLL1 inhibitor VTP50469 • Histone Deacetylase inhibitors • IDH1 and IDH2 inhibitors AG-221 and AG-120 • BET-bromodomain inhibitors
Chemotherapeutic agents	• CPX-351 • Vosaroxin • Nucleoside analogs
Mitochondrial inhibitors	• Bcl-2, Bcl-xL, and Mcl-1 inhibitors (Venetoclax) • Caseinolytic protease inhibitors
Antibodies and immunotherapies	• Monoclonal antibodies against CD33, CD44, • CD47, CD123, CLEC12A • Immunoconjugates (e.g., GO, SGN33A) • BiTEs and DARTs • CAR T cells or genetically engineered TCR • T cells • Immune checkpoint inhibitors (PD-1/PD-L1, • CTLA-4) • Anti-KIR antibody • Vaccines (e.g. WT1)
AML environment target therapies	• CXCR4 and CXCL12 antagonists • Antiangiogenic therapies

### Single cell analysis

NGS/single cell approaches allowed the improving of the risk classification of AML. It moved from the FAB morphological classification in favor of the molecular identity card of the ELN [51]. Compared to solid tumors, the number of mutations found in a given AML is much lower and several of them are druggable such as the *FLT3* mutations as well as *IDH1* and *IDH2*. Considering this, targeted therapies may be the ultimate weapon in the treatment of AMLs. Unfortunately, single cell approaches evidenced extreme heterogeneity of leukemic blasts that makes difficult the treatment of this pathology.

### Single cell RNA/DNA sequencing approaches

While out of the purposes of this review, some information about the methods used to profile single cell expression or DNA alterations may help readers in understanding the importance of these methods in the evaluation of AML heterogeneity, development, and identification of new therapies.

Single cell analyses start from the tissue dissociation to obtain single cells that can be compartmentalized and then analyzed. Tissue dissociation methods are based on mechanical, enzymatic, or a combination of both, dissociation methods to obtain high yields of viable single cells. Dissociation problem is simpler for tissues already dissociated in single cells as the blood.

Dissociated cells are then compartmentalized in small volumes to produce the sequencing library from each cell. Most promising methods for cell compartmentalization are based on microfluidics using nanowells (BD Rhapsody), microvalves (Fluidigim), and droplets (10X Genomics) [112]. The droplet based compartmentalization furnish the best throughput (about 10–30,000 cells analyzed per sample) and is becoming the gold standard for the analysis of single cells. For a discussion of other different methods used for cell compartmentalization see [113].

**Fig. 2:**
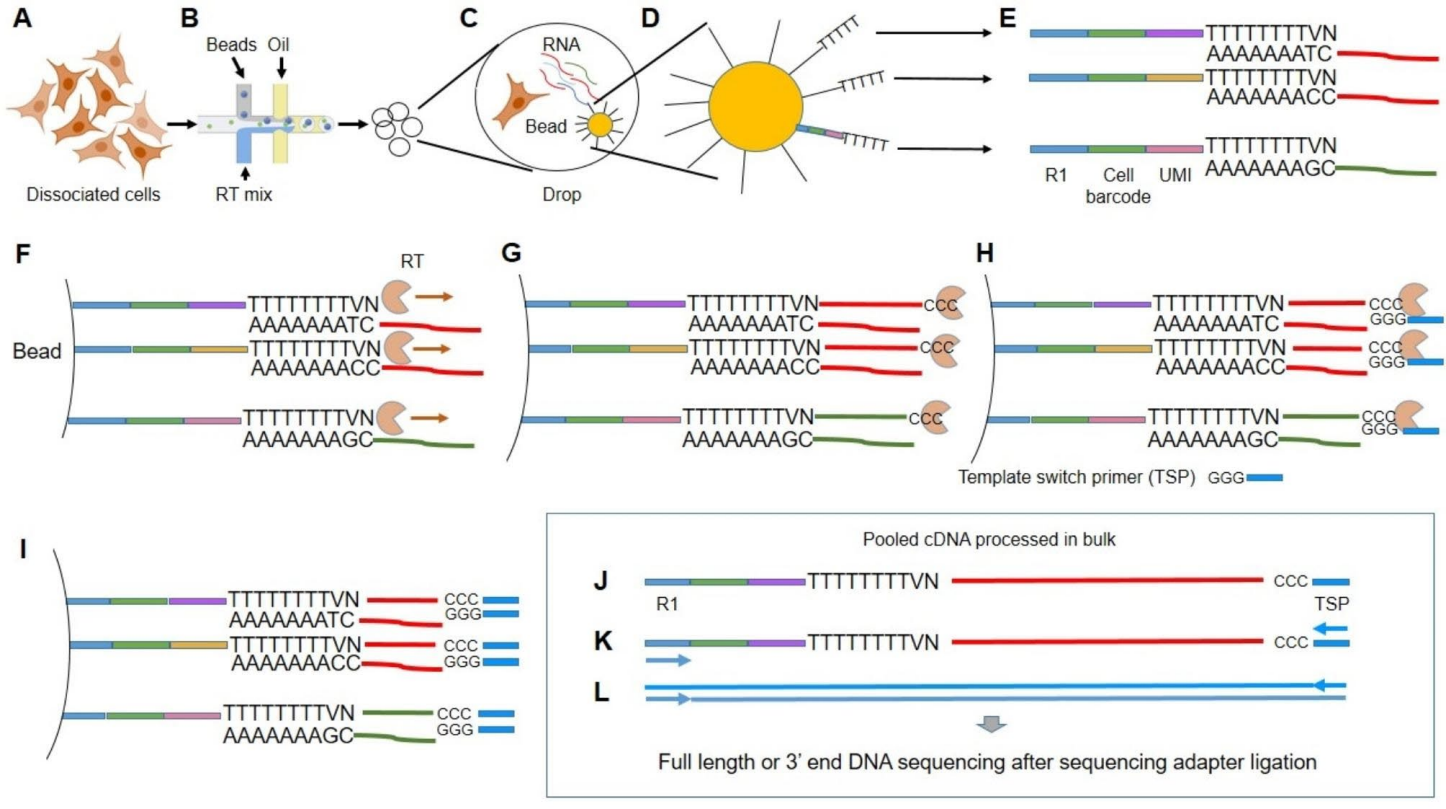
Description of the experimental process for RNA sequencing in single cells. A suspension of cells **(A)** is used to separate each cell into individual reactors. The figure describes the approach based on the formation of microbubbles **(B)**, which contain beads to allow the capture of RNA released by cell lysis within the reactor **(C)**. RNA capture by the bead in the reactor is permitted by covering the beads with oligonucleotides that have a T-tail in their 3’ terminal portion **(D)**. Each bead contains millions of these oligonucleotides that have common sequences (oligod(T) and R1), a unique sequence for each oligonucleotide (UMI) and a different sequence for each bead (cell barcode) **(E)**. The UMIs allow for the digital counting of gene expression because different UMIs can be associated with the same RNA sequence, based on the abundance of the RNA. The cell barcode allows understanding whether the RNAs being evaluated are derived from the same bead, and therefore cell, or from different beads, and therefore different cells. Oligod(T) allows the capture of polyadenylated RNAs and their retrotranscription **(F)**. Once retrotranscriptase (RT) reaches the 3’ terminal of RNA, due to its 3’ terminal transferase activity, it adds 3 cytosines to the cDNA **(G)**. The overhang of C allows binding of the template switch primer (TSP) and continuation of retrotranscription of this primer (H) as well. At the end of retrotranscription, the cDNA will be characterized by having known ends given by R1 and the template switch primer **(I and J)**. This whole process takes place within each reactor and therefore separately for each cell. Since at the end of the retrotranscription the cDNAs will be labeled with the cell barcode, it is possible to destroy the bubbles and continue the rest of the protocol in bulk. To amplify the low amount of RNA released from each individual cell, a PCR is done by exploiting primers complementary to R1 and TSP **(K)**. This will yield a dsDNA with two known ends **(L)** that can be used for the sequencing of the full length by binding sequencing adapters to the ends of cDNA by using a PCR or can be fragmented and sequenced the portion corresponding to the 3’ end of RNA.

Here we will explain the gold standard 10X Genomics for genomic and transcriptomic analyses at the single cell level. Droplet compartmentalization creates nanoreactors to retrotranscribe polyadenylated RNAs or to create DNA libraries using tagmentation (ATAC-seq). For the analysis of the RNA, cells are secluded with nanobeads that release within the droplet oligonucleotides composed by a stretch of thymidines to capture the mRNA and specific barcodes to recognize mRNA released from the cell within the droplet. Each oligonucleotide has a specific sequence (UMI) that is used to count the RNA. In fact, it is necessary to amplify the cDNA produced because the amount is insufficient for the next sequencing step. cDNA amplification is based on the SMART-PCR method [114] that in some cases preferentially amplify specific sequences. Therefore, if the aim of the experiment is to understand gene expression, it is important to avoid to base RNA quantification on the number of aligned reads to a specific gene. Using UMI it is possible to have a one-to-one relationship between mRNA and UMI also after the SMART-PCR because they are associated during the retrotranscription and before the PCR amplification (Fig. 2). If researchers are interested in the analysis of chromatin state, it is possible to use transposases within each droplet that target open chromatin allowing its fragmentation and insertion of sequencing primers (ATAC-seq). Finally, using antibody, it is possible to typing the cells basing on their surface proteins.

A problem of this technique is that, unfortunately, not all transcripts released from a single cell can be captured by the oligonucleotides that cover the bead within the microreactors. Therefore, to capture whole transcriptome for a specific call type it is necessary to sequence several types the same cell population and then cluster them basing on specific gene markers [115]. A key challenge in analyzing single cell RNA-sequencing data is the large number of false zeros, where genes actually expressed in a given cell are incorrectly measured as unexpressed. To overcome this problem several bioinformatics approaches were developed for data imputation [116–119] and allow following analyses (e.g. cell cluster, identification of differentially expressed genes).

### Improvements in AML comprehension thank to single cell analyses

*Single cell DNA sequencing.* The accumulation of somatic mutations within a tumor causes clonal diversity and is a consequence of therapeutic resistance, recurrence, and poor outcomes in cancer. A precise characterization of clonal diversity may help to move toward a personalized therapy. Single cell DNA sequencing (scDNA-seq) allows the precise discovering of clonal diversity within a tumor mass. Computational inference using variant allele fraction (VAF) data from massively parallel DNA sequencing of bulk tumor samples has been used to infer the clonal architecture of tumors [26, 120, 121]. However, the ability to infer clonal heterogeneity and tumor phylogeny from bulk sequencing data is inherently limited, because bulk sequencing techniques cannot reliably infer mutation co-occurrences and hence often fail in accurately reconstructing clonal substructure. Pellegrino and colleagues demonstrated the feasibility of scDNA-seq from AML samples taking advantage of cell compartmentalization by microfluidics [122]. The same research group profiled DNA sequences from single cells of 154 AML samples enabling them to reconstruct mutational histories [123]. The most frequently detected mutations by scDNA-seq were in *NPM1*, followed by *FLT3* (29% with internal tandem duplication (ITD) and 18% with non-ITD mutations), *DNMT3A, NRAS, IDH2, RUNX1, SRSF2, TET2*, and *KRAS*. scDNA-seq detected substantially more *FLT3* mutations than bulk-seq [123]. The authors demonstrated that using scDNA-seq it is possible to infer mutational history with a resolution not possible with bulk-seq. By simultaneous single-cell DNA and cell surface protein analysis, they illustrated both genetic and phenotypic evolution in AML. *NPM1* or *IDH* mutations were significantly associated with lower expression of CD34 and HLA-DR, whereas *TP53* mutations were associated with higher CD34 expression [123]. The simultaneous analysis of DNA and cell surface phenotypes by DAb-seq over multiple treatment time-points and disease recurrences demonstrated an extreme genotype-phenotype dynamics and the incongruity between blast cell genotype and phenotype in different clinical scenarios [124]. By analyzing samples after therapies (e.g. FLT3 inhibitor-containing or IDH2 inhibitor-containing) it is possible to evaluate changes in the tumor cell population (*123*). These results may sustain a continuous re-evaluation of the patient to identify alternative therapies that avoid relapse due to cell survive to the therapy. Knowing which cells are prone to survive to specific therapies by using a single cell approach, we can foresee the development of drugs to avoid relapse caused by surviving cells. ScDNA-seq can be performed also on specific portions of DNA. Targeted sequencing of mutational hotspots using 40 amplicons from 8 AML-specific genes and 16 time-points permitted to Peretz and colleagues to identify pathogenic variants not detected by clinical bulk NGS [125]. Again, the single cell approach evidenced polyclonal nature of the samples and the ability of the tumor to respond to treatments by originating new and previously undetectable mutations. Using scDNA-seq Peretz and colleagues demonstrated that RAS pathway mutations are a mechanism of clinical resistance to Quizartinib [125].

An example of the application of scDNA-seq and phenotyping of cell surface receptors was reported by Dillon and colleagues last year. The pairing of cell-surface immunophenotype with scDNA-seq genotyping allowed researchers to fully resolve the relationship between clonal hematopoiesis and AML in the three considered patients [126].

Genetic mutations associated with AML also occur in age-related clonal hematopoiesis, often in the same individual. This makes it difficult to safely assign the detected variants to malignancy. Therefore, it is important to identify functional alterations related with pathologies. In this context RNA sequencing appears to be useful.

*Single cell RNA sequencing.* Flow cytometry is widely used for exploring cell heterogeneity in leukemia; however, it is limited to the choice of surface markers [127]. RNA expression can be considered as a proxy of protein expression with the advantage respect to protein analysis of analyzing whole transcriptome because of the possibility of amplifying the signal using in vitro transcription [128] or SMART-PCR [114]. Single cell RNA-sequencing (scRNA-seq) is likely to become a method of increasing importance in the clinical diagnostic of hematologic malignancies [129]. Galen and colleagues used scRNA-seq to describe heterogeneity of leukemia cells [130]. They started describing normal BM-derived cells demonstrating that they can be separated in three major lineages of mature blood cells expressing established markers of hematopoietic populations (CD34 for HSC/Prog cells, CD14 for monocytes, and CD3 for T cells), as described in other manuscripts [131–135]. Performing the same analysis, Galen and colleagues did not distinguish normal and leukemia cells from their expression programs although they showed that cell type proportions markedly changed over the clinical course [130]. To distinguish tumor cells from normal cells the authors decided to sequence portions of transcripts that contain AML mutations from single cells and then profile their mRNAs. This method allows the identification of mutations in relation to gene expression (lower expressed genes are difficultly amplified) and in relation to the mutation positioning (those near to the 3’ end are more efficiently detected). The authors showed that mutations were not detected in healthy donors and were markedly decreased in AML patients in clinical remission meaning that cells with detected mutations were depleted from the patients. Interestingly, cells with mutations derived from myeloid axis and were classified as HSC-like, progenitor-like, GMP-like, promonocyte-like, monocyte-like, or cDC-like malignant cells.

Finally, the authors applied long reads obtained from MiniIon sequencing (Oxford Nanopore) to describe new tandem duplication and transcript fusion rearrangements demonstrating the feasibility of this approach also on single cells.

In this work the authors demonstrated that scRNA-seq data are consistent with clinical parameters but revealed more extensive malignant cell diversity than could be appreciated from a limited number of markers. Therefore, the approach provides more detailed information on AML cell types and differentiation states to deeper understand the better treatment to contrast the progression of the pathology.

The integration of scRNA-seq and scDNA-seq permitted to Petti and colleagues[136] to demonstrate that expression heterogeneity relates to subclonal genetic events: a particular expression of a cell correspond to mutationally defined somatic mutations. The same authors also stated that the data revealed transcriptional heterogeneity that occurred independently of subclonal mutations, suggesting that additional factors drive epigenetic heterogeneity. Apparently from this study resulted particularly important the integration of different omics information to better understand the heterogeneity in gene expression patterns.

Another study conducted on 40 de novo AMLs showed that cell groups were less distinct when compared with those of healthy BM with the lack of cell cluster boundaries [137]. In this study The authors showcased that a high expression levels of ribosomal proteins in AML progenitor cells is a predictor of poor prognosis providing a new perspective for the classification of AML or to evaluate the effects of the therapy.

Lefort and colleagues reported that the BMP pathway sustains a permanent pool of LSCs that express high levels of BMPR1B receptor and, upon treatment, evolve to progressively implement an autocrine loop of BMP4, leading to cells resistant to tyrosine kinase inhibitors (TKIs). RNA-Seq analysis of single TKI-resistant LSCs revealed co-activation of the Smad1/5/8 and Stat3 pathways, which could be targeted by blocking BMPR1B/Jak2 signaling. A specific inhibitor of BMPR1B impaired BMP4-mediated protection of LSCs against TKIs [138]. Targeting BMPs could eliminate leukemic cells within a protective BM microenvironment to effectively affect residual resistance or persistence of LSCs in myeloid leukemia. This work sustains the possibility of identifying specific pathways in resistant cells that can be targeted also by already used drugs to better treat patients.

It is known that tumor microenvironment plays an important role in tumor progression and response to therapy. Several studies have used scRNA-seq to investigate tumor microenvironment and immune response in AML. For example, Zhang and colleagues [139] used scRNA-seq data from 128,688 cells to reveal the microenvironment and exhaustion status of T/NK cells in AML. Specific gene expression associated with T/NK cell exhaustion was identified, providing insight into immune dysfunction in AML. Furthermore, Pan and colleagues [140] performed a comprehensive scRNA-seq study in AML samples to identify patterns of immune response to tumor microenvironment, prognosis, and immunotherapy response This study highlights the importance of of aggrephagy-related patterns in tumor microenvironment. It is a kind of selective autophagy to clear protein aggregates. Once the function of molecular chaperone and ubiquitin proteasome is limited or the clearance efficiency of misfolded proteins is lower than the production rate, protein aggregates, and the aggrephagy needs to be activated to degrade them.

Single cell RNA sequencing of bone marrow cells from AML patients allowed the identification of a novel microRNA: hsa-miR-12,462 [141]. They found that overexpression of hsa-miR-12,462 in AML cells significantly decreased their growth rate. This highlights the importance of epigenetic alterations that were discussed also by Duchmann and colleagues [142].

After medical treatments it is possible that some cancer cells remain in the body. Since they are fundamental for relapse it is important to understand if they are present. Minimal residual disease (MRD) refers to the small number of cancer cells that remain in the body after treatment. Their identification is difficult because they do not cause any physical signs or symptoms and often cannot even be detected through traditional methods such as viewing cells under a microscope and/or by tracking abnormal serum proteins in the blood.

Dillon and colleagues developed a targeted RNA-sequencing-based approach for the quantification of MRD in AML using scRNA-seq [143] that permitted to detect all newly approved European Leukemia Network molecular targets for MRD in AML, with a limit of detection as low as 1 in 100,000 cells. This result is comparable to the flow cytometry that usually bases the detection to a reduced number of surface markers. Alternatively, with PCR, it is possible to identify one cancer cell within 100,000 to one million normal cells and next generation sequencing can detect one cancer cell in one million bone marrow cells checked.

*Spatial transcriptomic.* Lefort and colleagues showed the importance of protective BM microenvironment against the treatment of leukemia [138]. For a review describing the BM microenvironment mechanisms in AML see [144]. Here we want to highlight the fact that the analysis of single cells is based on the loss of cellular interactions and thus the loss of information related to cell-cell relationship and communication. Latterly, the spatial transcriptomic was applied to evaluate alterations in BM. Spatial transcriptomic allows the evaluation of gene expression maintaining tissue structure. It allows RNA sequencing or highlight specific transcripts on a tissue slice [145]. Recently transcriptional map all major BM-resident cell types and their spatial allocation to distinct BM niches was described by Baccin and collaborator [146]. The authors showed that arteriolar and sinusoidal vascular scaffolds represent key sites for the production of factors required for HSC maintenance and differentiation. These sites have central hubs populated with previously unappreciated subpopulations of cells named by the authors Adipo- and Osteo-CAR cells. The model proposed by the authors sustains that the establishment of distinct niches is mediated by the differential localization of professional cytokine-producing cells.

ScRNA-seq provided an improvement in understanding the cellular ecosystem of both leukemic and residual normal hematopoietic cells not possible until a few years ago with bulk RNA sequencing [130, 147].

### CAR-T and genomic tools to improve the treatment of AML

With the advent of single cell RNA sequencing it is possible to evidence subpopulation of tumor cells also after the treatment with conventional chemotherapies. This allows to identify their surface antigens that may be recognized by specific immune cells. In this context the immunotherapy techniques may take advantages for the treatment of AML. They are based on vaccine therapy, monoclonal antibodies, checkpoint inhibitors, stem cell transplantation, and Chimeric Antigen Receptor (CAR) T cell therapy. These therapies are discussed in the Winer and Stone’s review [148]. Here we will discuss how the recently advent of CAR-T cell therapy may take advantage from information retrieved from single cell RNA sequencing to avoid relapse or to ameliorate the treatment of AML. CAR-T cell therapies were approved by FDA in 2017 and transformed the treatment of blood cancers. By providing immune cells with the information they need to better recognize cancer cells as foreign and attack them, this type of targeted immunotherapy aims to boost the immune system. The production of CAR-T cells is well described in [149].

CAR-T cell therapies have made great strides against B-cell-derived leukemias and lymphomas, but have been largely ineffective against myeloid cell-derived leukemias. In fact, CAR-T can target not only cancer cells but also normal cells. This is not a judge problem with lymphoid malignancies, such as acute lymphoblastic leukemia and B-cell lymphomas, because diminished ability to produce immunoglobulins can be compensated by replacing them with transfusions. Differently, the elimination of normal myeloid cells affects the ability of the body to respond to infections. Before the study published from Leick and colleagues, CAR-T therapy of AML has been hampered by the lack of suitable antigens and by off-target effects. They identified CD70 as an antigen largely present in AML cells. In a recently published work the team demonstrated that to improve CAR-T therapy of AML condition it is necessary to combine it with drug therapy based on azacitidine. It has the ability to improve the number of CD70 antigens on the surface of cancer cells. Leick and colleagues also engineered CAR-T cells to improve the strength and durability of the tumor-killing effect by stabilizing the binding of the CAR to CD70 [150].

CD70 is not the only antigen against CAR-T cells may work in AML fighting. Table 6 summarizes different clinical trials based on CAR-T.

CD33 is a transmembrane protein of the sialic acid-binding immunoglobulin-like lectin (SIGLEC) family. CD33 is expressed in normal progenitor cells, myeloid cells, and more than 90% of AML cells and has diagnostic and therapeutic capabilities. The recombinant humanized anti-CD33 antibody conjugated to calicheamicin (gemtuzumab ozogamicin; GO) is used for the treatment of AML as the only approved drug. This supports the potential of using CD33 as a target antigen against which CAR-T cells can act. Some in vivo and in vitro studies have demonstrated the antitumor activity of CD33-CAR-T cells against AML cells [151–156].

CD38 is a type II transmembrane glycoprotein that is expressed in AML blasts but not in normal human hematopoietic stem cells. To engineer CD38-CAR-T cells against AML cells, the intensity and number of CD38 should be increased. All-trans retinoic acid (ATRA) as a therapeutic factor for the treatment of acute promyelocytic leukemia has the ability to induce CD38 expression in AML cells. It was observed that CD38-CAR-T cell combined with ATRA eliminate the tumor cells [157].

The IL-3 receptor α subunit (IL3Rα) is named CD123 and is overexpressed on leukemic stem cells (LSCs) and AML blasts with no significant expression on normal hematopoietic stem cells. This makes it as a potential therapeutic target but an anti-CD123 neutralizing monoclonal antibody demonstrated insufficient efficacy against AML [158]. CD123-CAR-T cells with ScFv composed of VL and VH from various mAbs presented the low off-tumor toxicity and lysis effect on healthy hematopoietic stem cells compared to CAR-T cells with VL and VH chains of only one mAb [159].

C-type lectin-like molecule-1 (CLL1) is a type II transmembrane glycoprotein overexpressed on differentiated myeloid cells and AML blasts in 92% of AML cases. This allowed to produce monoclonal Ab against CLL1 as well as CLL1-CAR-T cells with an effective therapeutic function against AML along with reducing tumor burden [160].

Lewis Y (LeY) is a carbohydrate tumor-associated antigen related to blood-group members. Due to LeY expression on early myeloid progenitor cells, it can be a proper targeting choice for AML treatment [161]. LeY-CAR-T demonstrated cytolytic response against LeY + tumor cells and high-level production of IFN-γ [162].

Wilms Tumor 1 (WT1) is a zinc-finger transcription factor overexpressed in various hematological disorders [163]. WT1-CAR-T cells were used against WT1+/HLA-A*02:01 + primary tumor cells or cell lines with satisfactory results [164].

CD7 is a transmembrane glycoprotein expressed by leukemic cells like AML (30%) but not by healthy myeloid cells [165]. Due to CD7 expression on T cells it is necessary to engineering CAR-T cells to avoid CD7 expression ad allow targeting of only tumor cells. Reduction of tumor burden indicated that CD7-CAR-T cell prevents systemic leukemia progression making them treatment for refractory or relapsed AML [166, 167].

Natural killer group 2D (NKG2D) is a receptor whose ligands are overexpressed in various hematological malignancies [168]. Autologous first-generation NKG2D-CD3ζ-CAR-T cells were developed by Baumeister et al. [169]. They validated CAR-T cells in AML patients evidencing in one of these a clinical response with a high level of IFN-γ production.

CAR-T cells are important weapons against tumors, but their proliferation, persistence, and anti-tumor functions may decrease encountering some challenges in hematological malignancies. Delivery methods impact on CAR-T activity with the local delivery better than systemic one [170, 171]. In vitro proliferation of CAR-T cells is another problem in the production of these therapeutic compounds also considering the small starting amount of T cells due to treatments that patients have to manage the tumor. Persistence, proliferation, and efficacy of CAR-T are features that researchers have to improve constantly. The incorporation in CAR-T structure of co-stimulatory receptor is a prosperous manner of overcoming poor persistence [172–176]. CAR-T cells may induce side effects like abundant production of cytokines, off target toxicity, anaphylaxis because the presence of murine ScFv, neurotoxicity, and insertional oncogenesis due to lentiviral or retroviral activity. In fact, the production of CAR-T cells is based on lentiviral or retroviral transfection to deliver the CAR gene into T cells. This cause its random insertion in the genome of the host cells potentially causing unwanted genetic side effects.

**Fig. 3:**
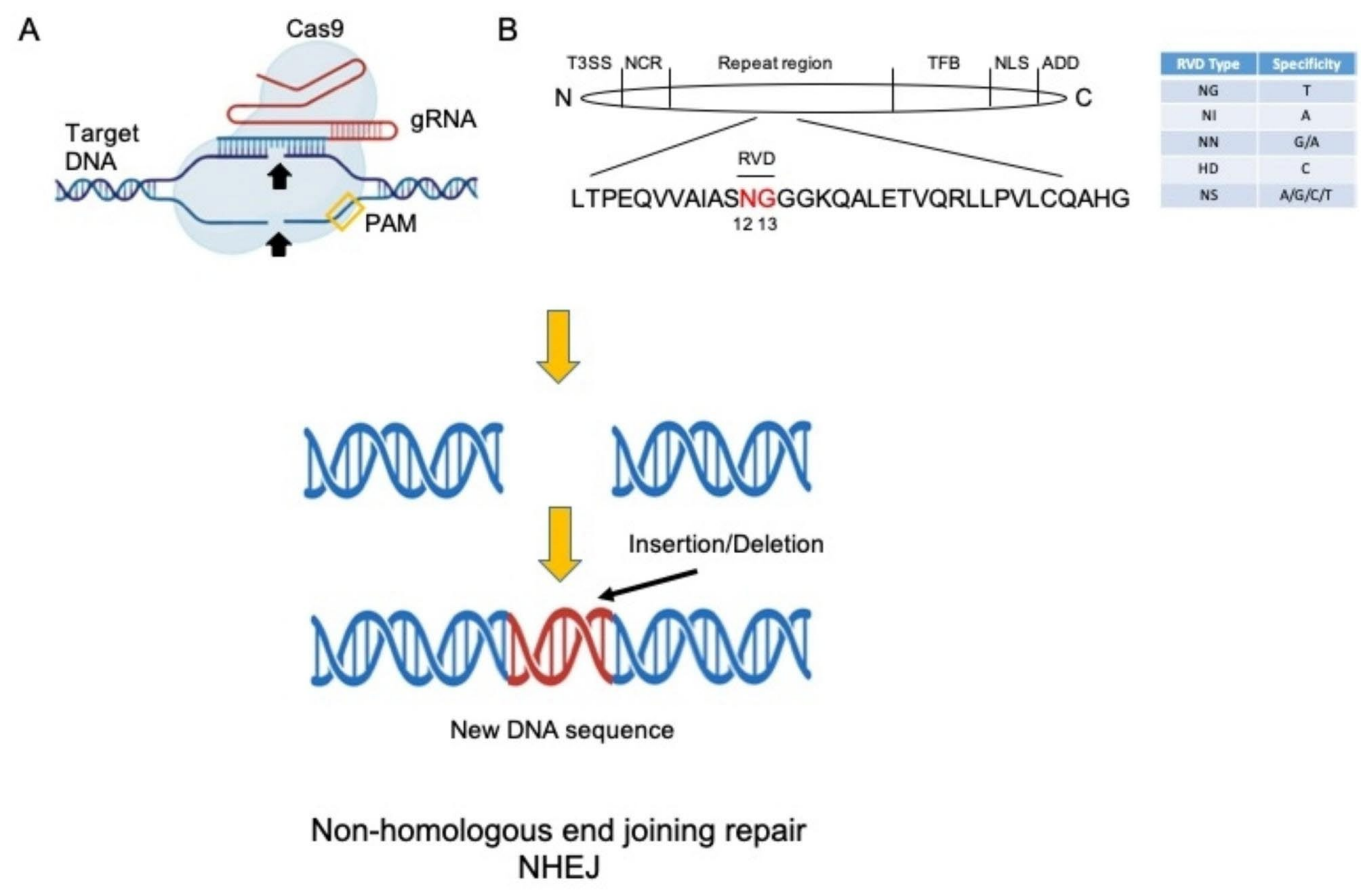
Description of gene editing. **(A)** Cas9 is a protein that can induce double-stranded brakes in a target DNA (blue in the figure). Cas9 is guided at the DNA site through an RNA guide (gRNA) that has a proper 3D conformation and a complementary to a DNA sequence near a PAM motive that is different for different Cas types. **(B)** Architecture of TALEs. The N-terminal region of a TALE contains a type III secretion signal (T3SS) and four non-canonical repeats (NCR), the C-terminal part a transcription factor binding site (TFB), two nuclear localisation signals (NLS) and an acidic activation domain (AAD). Each TALE also contains a repeat region with a variable number of highly conserved 33-35-aa repeats arranged in tandem. The amino acid sequence of a consensus 34-aa TALE repeat is shown and the amino acids responsible for the DNA specificity of a TALE, the variable repeat di-residues (RVDs), are highlighted. The five most commonly used RVDs and the nucleotides they specify are also shown in the table. In both cases a double strand break in the target DNA sequence is induced. The cell activate repair mechanisms that cause the introduction of an insertion or a deletion at the site where the DNA was broken. If the site is in a coding sequence, the protein will not be produced because the introduction of wrong codon stops

Currently CAR-T therapies have some limitations (suboptimal T cell quality when isolated from patients who have already been treated with lymphotoxic chemotherapies, poor CAR-T cell persistence, economic barriers for production, quality control of patient-derived CAR-T cells, long time to infusion of CAR-T cells which may reduce overall outcome) and researchers, to augment their efficacy and overcome limitations, have turned to gene-editing technologies such as CRISPR-Cas, transcription activator-like effector nucleases (TALENs) and meganucleaases. The discovery of meganucleases (naturally occurring restriction enzymes that can recognize 12–40 bp DNA sequences) was the initial step towards genome editing, followed by the discovery of zinc finger nucleases (ZFN) in the 1980s [177] CRISPR/Cas9 is the most recent developed robust genome-editing tool with high precision (Fig. 3A) that can substitute lenti- or retro-viruses for the genome modification of T cells [178]. Until now, it was not clinically tested CAR-T energized with CRISPR/CAS9 technique in the AML field probably because it relatively recent development (2012) in comparison with TALEN approach. In fact, phase I clinical trials (NCT01864902, NCT03190278) are ongoing for testing CAR-T cells engineered with TALEN approach [179], developed in 2010, are fusions of a transcription activator-like effector (TALE) and the catalytic domain of the restriction endonuclease FokI. Transcription activator-like effectors (TALEs) are proteins from plant pathogenic *Xanthomonas* bacteria. TALE proteins contain three functional domains; the key domain for their specific and programmable DNA-binding is a central repeat region composed of tandem repeats of amino acids where position 12 and 13 of each repeat define the DNA binding specificity (Fig. 3B). Endonuclease associated with TALE produces a double strand DNA (dsDNA) break that is processed as in the case of dsDNA break produced by CRISPR/CAS9 tool (Fig. 3). NCT01864902, NCT03190278 clinical trials aim at evaluating the safety and efficacy of CAR-T targeting CD123 in patients with relapsed/refractory AML. TALENS are used to disable the TCRαβ gene T cells use to recognize ’self’ to prevent them from attacking the host.

Limitations in the usage of genomic tools to manipulate the DNA of cells are associated with the frequency of induced mutation and their specificity. It has been reported levels of over 50% of mutations using the best TALEN and CRIPR/CAS9 systems [179, 180]. Several factors affect the off-target effect generated by a nuclease: (a) the frequency of the homologous sequence in the genome, (b) the level of nuclease expression, (c) the duration of nuclease exposure, (d) target site accessibility. This aspect is well discussed in [181]. There are limited paper that identified no off targets of nucleases and several depend on the approach used to identify off targets, but the development of engineered DNA cutting proteins allowed to produce limited effects on off target genomic regions.

**Table 6:** CAR-T cell based therapies. R/R relapsed/refractory, AML acute myeloid leukemia, ALL acute lymphoblastic leukemia, MDS myelodysplastic syndrome, CML chronic myeloid leukemia, MPN myeloproliferative neoplasm, alloHSCT allogeneic hematopoietic stem cell transplantation, RAEB refractory anemia with excess blasts, MM multiple myeloma

Target antigen	Clinical trial ID	Phase	Disease
**CD33**	NCT03126864	I	R/R AML
	NCT02799680	I	R/R AML
	NCT01864902	I/II	R/R AML
	NCT02944162	I/II	R/R AML
	NCT03291444	I	R/R AML, MDS; ALL
	NCT03473457	n.a.	R/R AML
	NCT03222674	I/II	AML
**CD38**	NCT03291444	I	R/R AML, MDS; ALL
	NCT03473457	n.a	R/R AML
	NCT03222674	I/II	AML
**CD123**	NCT03585517	I	AML
	NCT03114670	I	Recurred AML after alloHSCT
	NCT03556982	I/II	R/R AML
	NCT02623582	I	R/R AML
	NCT02159495	I	R/R AML
	NCT03672851	I	R/R AML
	NCT03766126	I	R/R AML
	NCT03291444	I	R/R AML, MDS; ALL
	NCT03473457	n.a	R/R AML
	NCT03796390	I	R/R AML
	NCT03222674	I/II	AML
**UCART123**	NCT03190278	I	R/R AML
	NCT01864902	I	R/R AML, high-risk AML
**CD123/CLL1**	NCT03631576	II/III	R/R AML
**CD33/CLL1**	NCT03795779	I	R/R AML, MDS, MPN, CML
**CCL1**	NCT03222674	I/II	AML
**Lewis Y**	NCT01716364	I	Myeloma, AML, MDS
**WT1**	NCT03291444	I	R/R AML, ALL, MDS
**CD7/NK92**	NCT03018405	I/II	R/R AML
**NKG2D**	NCT02203825	I	AML, MDS-RAEB, MM
	NCT03018405	I/II	R/R AML, AML, Myeloma

## Conclusions

Already the philosophers of Ionian Greece of the 7th century B.C. they conceived the current of thought called Atomism assuming a plurality of fundamental constituents at the origin of physical matter, which would tend to aggregate and disrupt.

In the history of medicine and biology, immense strides have been made in the understanding of the functionality and architecture of the human body. Above all in the last century the progression of science and knowledge had a sudden climb providing the world with sensational discoveries in ever shorter periods of time: in 1953 the structure of DNA [182], DNA sequencing in 1977 [183, 184] in 1984 the PCR molecular technique which completely carried out the genetic investigations [185] in 2003 the sequencing of the human genome [186], in 2008 the high throughput NGS methods [187–189], in the 10s of the new millennium the advent of single cell analysis techniques [112], in 2014 the innovative gene editing technology CRSPR/CAS9 [190].

It is therefore now clear how the ancient thought of the Atomists can approach the reality of the biology and pathology of the tissues of the human body. Returning to the Lewis Caroll quotation with which we started this paper, “it’s a great puzzle”, the large and complicated puzzle that represents the tumour heterogeneity that characterizes, in this case AML, can be completed thanks to the advent of the new single cell investigation techniques.

Several studies have investigated genome or transcriptome alterations of r/r AML. These analyses provided an exceptional contribution in solving the clonal evolution of the disease, characterizing its molecular patterns, thus allowing us to think about target therapy.

The next step is to exploit single-cell investigation techniques such as scRNA-seq to be able to discriminate the clonal and subclonal composition of the tumor as well as the identification of cells causing relapse after chemotherapy treatments.

The vast panorama of cell-by-cell investigations ranging from proteomics (cytofluorimetry) to genomics (scDNA-seq) and transcriptomics (scRNA-seq) has allowed, and will increasingly allow in the future, to discern the various components of the puzzle represented by the subclonal cellular components constituting the tumor.

The FAB, WHO and ELN characterization of the molecular profiles of AMLs has allowed us to stratify the risk of diagnosis and prognosis of these terrible haematological tumours. Increasingly in-depth study and the discovery of further pieces of the puzzle have made it possible to highlight therapeutic targets and therefore the possibility of creating new directed drugs by limiting side effects as much as possible and ameliorating the prognosis. The development target drugs, antibodies and CAR-T treatments is also crucial, for which there are great expectations for the future.

Technological innovation regarding the so-called omics sciences has the massive power to outline a molecular identity card of human body allowing, when necessary, to customize drug therapy or treatment following the development of diseases.

## Data Availability

The data generated are included within the manuscript.
